# Expression of tripartite motif-containing 44 and its prognostic and clinicopathological value in human malignancies:a meta-analysis

**DOI:** 10.1186/s12885-020-07014-w

**Published:** 2020-06-05

**Authors:** Guoliang Xiao, Qiuxi Yang, Ziwei Bao, Haixia Mao, Yi Zhang, Shibu Lin

**Affiliations:** 1 Department of General Surgery, the First People’s Hospital of Neijiang, Neijiang, 641000 Sichuan Province PR China; 2 Department of Hepatobiliary Surgery, The First Affiliated Hospital of Hainan Medical University, Haikou, 570100 Hainan Province PR China; 3 Department of medicine, Southwest Medical University, Luzhou, 646000 Sichuan Province, PR China

**Keywords:** TRIM44, Prognosis, Clinicopathological features, Meta-analysis

## Abstract

**Background:**

Previous researches have reported that tripartite motif-containing 44 (TRIM44) is related to the prognosis of multiple human tumors. This study was designed to systematically assess the prognostic value of TRIM44 in human malignancies and summarize its possible tumor-related mechanisms.

**Methods:**

The available databases were searched for eligible studies that evaluated the clinicopathological and prognostic roles of TRIM44 in patients with malignancies. The hazard ratios (HR) and odds ratios (OR) were combined to assess the predictive role of TRIM44 using Stata/SE 14.1 software.

**Results:**

A total of 1740 patients from thirteen original studies were finally included in this study. The results of the combined analysis showed that over-expression of TRIM44 protein was significantly correlated with shorter overall survival (OS) (HR = 1.94, 95% CI: 1.60–2.35) and worse disease-free survival (DFS) (HR = 2.13, 95% CI: 1.24–3.65) in cancer patients. Additionally, the combined ORs indicated that elevated expression level of TRIM44 protein was significantly associated with lymph node metastasis (OR = 2.69, 95% CI: 1.71–4.24), distant metastasis (OR = 10.35, 95% CI: 1.01–106.24), poor tumor differentiation (OR = 1.78, 95% CI: 1.03–3.09), increased depth of tumor invasion (OR = 2.72, 95% CI: 1.73–4.30), advanced clinical stage (OR = 2.75, 95% CI: 2.04–3.71), and recurrence (OR = 2.30, 95% CI: 1.34–3.95). Furthermore, analysis results using Gene Expression Profiling Interactive Analysis (GEPIA) showed that the expression level of TRIM44 mRNA was higher in most tumor tissues than in the corresponding normal tissues, and the relationship between TRIM44 mRNA level and prognosis in various malignant tumors also explored in GEPIA and OS analysis webservers.

**Conclusions:**

TRIM44 may serve as a valuable prognostic biomarker and a potential therapeutic target for patients with malignancies.

## Background

Malignant cancers are one of the main causes of disease-related death in the world. Advances in detection techniques have contributed to the discovery of early cancer, and the progress of perioperative treatment has reduced the mortality in patients with malignancies. However, after standard treatment, patients with advanced malignancies still show poor survival rates due to consequent recurrence or metastasis. Due to the ongoing intensive cancer research, the precise tumor-related mechanisms have gradually been understood and an increasing number of therapeutic targets have been identified.

The field is moving toward targeted therapy as a primary form of tumor therapy. Therefore, major efforts have been made to identify molecular markers that predict the prognosis. More importantly, these markers can often be used as therapeutic targets, and then the corresponding targeted drug design can be carried out. Compared with gene therapy, the current targeted drug design for functional proteins may be more conducive to bringing new progress in tumor therapy.

Recent studies have shown that some tripartite motif (TRIM) proteins are involved in tumorigenesis and progression, and they function as protein regulators, and involve in important intersections in the gene pathways [1]. Tripartite motif-containing 44 (TRIM44) localized in the cytoplasmic compartment of cells was reported to contribute to diverse pathological conditions, such as tumors, growth disorders, and neurodegeneration [2–6]. TRIM44 protein has a zinc-finger domain, which plays the role of ubiquitin-specific proteases (USPs). Thus, it has been defined as the “USP-like” TRIM [6]. The ubiquitin–proteasome system plays an important role in the regulation of cell function and is the intersection of multiple regulatory pathways [7]. TRIM44 is an atypical TRIM-family protein that lacks the RING-finger domain but contains a zinc-finger domain that is often found in ubiquitin-specific proteases [8].

Some studies have suggested that TRIM44 plays a cancer-promoting role in oncogenesis and tumor progression, and increased TRIM44 expression was detected in cancer tissues and it was associated with poor prognosis and advanced clinicopathological parameters [9–11]. Importantly, recent studies have revealed that high levels of TRIM44 induce the epithelial-to-mesenchymal transition (EMT) in cancer cells and that TRIM44 promotes tumor initiation and progression by activating the PI3K/AKT/mTOR pathway [10, 12]. Another report indicated that TRIM44 could activate the NF-κB pathway to promote lung cancer cell migration and invasion [13].

However, to date, no specific meta-analysis has been performed to evaluate the association between TRIM44 expression and clinical outcomes in diverse malignancies. Therefore, we conducted this study to provide a systematic evaluation of the predictive value of TRIM44 and explore its feasibility as a new therapeutic target.

## Methods

### Search strategy

A comprehensive search strategy was conducted in the following databases: Web of Science, PubMed, CNKI, Wanfang, EMBASE, and Google Scholar. The search deadline was November 2, 2019. The following keywords were adopted according to the retrieval strategy: “Tripartite motif-containing 44” OR “TRIM44”; “cancer” OR “tumor” OR “carcinoma” OR “malignancy.”

### Inclusion and exclusion criteria

Inclusion criteria were as follows: 1) The expression of TRIM44 protein was measured in tissue samples from primary solid cancers; 2) All patients included in the original studies were divided into two groups according to the expression levels of TRIM44 protein; 3) The hazard ratio (HR) of survival outcomes or clinicopathological data based on the high and low levels of TRIM44 protein expression was reported; 4) The survival curve presented or sufficient data were available for calculating the HR with 95% CI.

Exclusion criteria were as follows: not original studies, studies without a control group, articles that only explored the molecular functions of TRIM44, and studies with less than 50 cases.

### Data extraction

For each study, the following general information was independently collected by two investigators (ZWB and HXM): author’s name, number of patients, cancer type, high expression rate, end-points (analysis type), evaluation standard of TRIM44 overexpression, follow-up time, detection method, and outcome measures.

Additionally, information on the clinicopathological parameters related to tumor progression was collected. For survival data extraction, the HRs and 95% CIs were directly used from the multivariate survival analysis or the univariate analysis second priority; otherwise, they were retrieved using the Engauge Digitizer version 4.1 if a study only provided Kaplan-Meier curves.

### Quality assessment

Newcastle-Ottawa quality assessment scale (NOS) was adopted to evaluate the quality of enrolled studies. This method comprised three parameters of quality: selection (score: 0–4), comparability (score: 0–2), and outcome assessment (score: 0–3), with total scores ranging from 0 to 9 [14]. The study with total scores greater than 6 was considered high quality in the present meta-analysis.

### Public data and tools

In this study, the Gene Expression Profiling Interactive Analysis (GEPIA) database (http://gepia.cancer-pku.cn/), an online database containing RNA expression information and survival data from the TCGA and the GTEx projects, was utilized [15]. This database consists of 33 different types of human malignant tumors, including ACC (Adrenocortical carcinoma), BLCA (Bladder urothelial carcinoma), BRCA (Breast invasive carcinoma), CESC (Cervical squamous cell carcinoma and endocervical adenocarcinoma), CHOL (Cholangio carcinoma), COAD (Colon adenocarcinoma), DLBC (Lymphoid neoplasm diffuse large B-cell lymphoma),

ESCA (Esophageal carcinoma), GBM (Glioblastoma multiforme), HNSC (Head and neck squamous cell carcinoma), KICH (Kidney chromophobe), KIRC (Kidney renal clear cell carcinoma), KIRP (Kidney renal papillary cell carcinoma), LAML (Acute myeloid leukemia), LGG (Brain lower grade glioma), LIHC (Liver hepatocellular carcinoma), LUAD (Lung adenocarcinoma), LUSC (Lung squamous cell carcinoma), MESO (Mesothelioma), OV (Ovarian serous cystadenocarcinoma), PAA (Pancreatic adenocarcinoma), PCPG (Pheochromocytoma and paraganglioma), PRAD (Prostate adenocarcinoma), READ (Rectum adenocarcinoma), SARC (Sarcoma), SKCM (Skin cutaneous melanoma), STAD (Stomach adenocarcinoma), TGCT (Testicular germ cell tumors), THCA (Thyroid carcinoma), THYM (Thymoma), UCEC (Uterine corpus endometrial carcinoma), UCS (Uterine carcinosarcoma) and.

UVM (Uveal melanoma).

Here, GEPIA was used to show the expression level and the prognostic value of TRIM44 in various human cancers. A box plot was used to represent the RNA expression level in samples. And patients were divided into high-expression and low-expression groups according to the median expression level of TRIM44, and the Kaplan-Meier plots for survival analysis were performed.

Besides, the prognostic value of TRIM44 in the specific type of cancer was explored by some online OS analysis webservers, including OSgbm for glioblastoma [16], OSpaad for pancreatic carcinoma [17], OSbrca for breast cancer [18], OSacc for adrenocortical carcinoma [19], OSuvm for uveal melanoma [20], OScc for cervical cancer [21], OSkirc for kidney renal clear cell carcinoma [22], OSescc for esophageal squamous cell carcinoma [23], OSblca for bladder cancer [24], OSlms for leiomyosarcoma [25].

### Statistical analysis

STATA/SE 14.1 was used to analyze the relationship between TRIM44 expression and prognosis as well as the clinicopathological features in human cancers. I^2^ statistics and Chi-square Q test were adopted to estimate the heterogeneity among enrolled studies. The random-effects model was adopted to conduct the meta-analysis with significant heterogeneity (I^2^ ≥ 50% or P_Q_ < 0.10), while the fixed-effects model was used when there was no obvious heterogeneity (I^2^ < 50% or P_Q_ ≥ 0.10). The funnel plot and Begg’s test were applied to assess publication bias, and sensitivity analysis was used to test the stability of the analysis results. *p* < 0.05 indicates a significant difference in this study.

## Results

### Characteristics of eligible studies

**Fig. 1:**
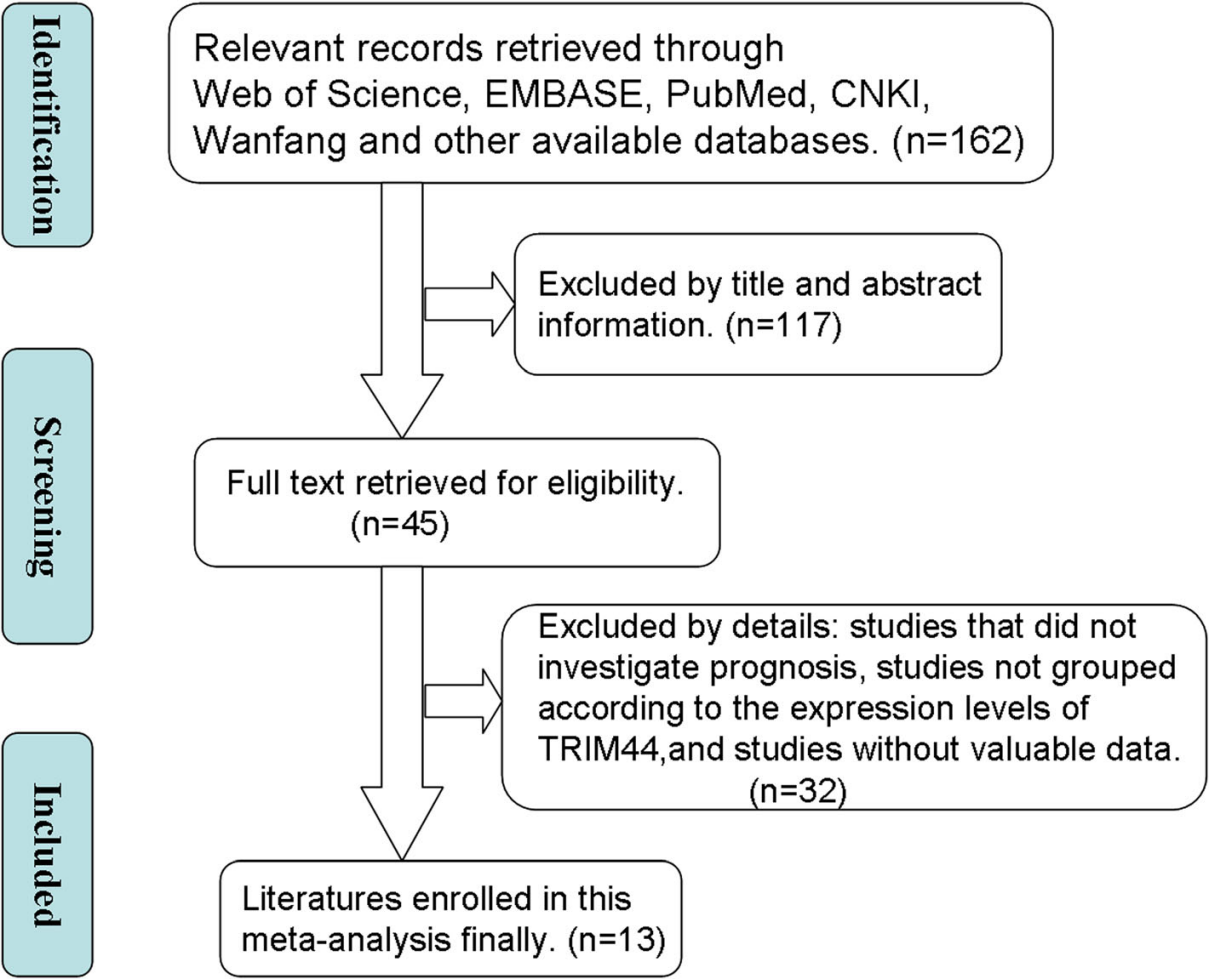
Flowchart depicting the steps of the literature search and selection process

Study collection and screening process are shown in Fig. 1. After further discussion and consideration of the selected articles, 13 cohort studies published between 2012 and 2019 were selected for further analysis [9, 10, 26–36]. In total, these 13 studies included 1740 patients with a mean sample size of 133.8 and a range from 68 to 331. Eleven studies presented data on the association between the TRIM44 protein levels and OS, and four studies presented data on the link between TRIM44 and DFS. In these studies, 12 different kinds of solid tumors were analyzed, including gastric cancer (GC), osteosarcoma, cervical cancer (CC), breast cancer (BC), hepatocellular carcinoma (HCC), endometrial carcinoma (EMC), esophageal cancer (EC), melanoma, testicular germ cell tumor (TGCT), non-small cell lung cancer (NSCLC), intrahepatic cholangiocarcinoma (ICC), and epithelial ovarian cancer (EOC). All primary cancer tissues and adjacent non-tumor tissue samples were collected from patients in Japan and P.R. China. The expression of TRIM44 protein in tissue samples was measured by immunohistochemistry (IHC). All included articles were written in English, and they were of good quality. The basic characteristic information is presented in Table 1.

**Table 1:** Characteristics of included studies

Author	Year	Disease	Country	Sample size	Over-expression (N, %)	Detection method	Evaluation standard of TRIM44 overexpression	End-points (analysis type)	Follow-up	NOS score
Kashimoto K	2012	GC	Japan	112	28, 25%	IHC	Final staining scores^a^ ≥ 4	OS (M)	NR	8
Kawabata H	2017	BC	Japan	129	67, 52%	IHC	Final staining scores^a^ ≥ 5	OS (M)	≥ 5 years	9
Kawaguchi T	2017	EC	Japan	68	39, 57%	IHC	Final staining scores^b^ ≥ 1	OS (M)	≥ 5 years	9
Li P	2018	EMC	China	143	80, 56%	IHC	Final staining scores^a^ ≥ 4	OS (C)	≥ 5 years	7
Liu S	2018	EOC	China	109	93, 85%	IHC	Final staining scores^a^ ≥ 3	OS (M), DFS (M)	≥ 5 years	7
Peng R	2018	ICC	China	130	71, 55%	IHC	Percentage of TRIM44 positive cells ≥50%	OS (M)	≥ 5 years	8
Xing Y	2016	NSCLC	China	331	208, 63%	IHC	Final staining scores^c^ ≥ 4	OS (M), DFS (M)	≥ 5 years	9
Xiong D	2018	EC	China	100	53, 53%	IHC	Final staining scores^c^ ≥ 4	OS (M)	≥ 5 years	8
Yamada Y	2017	TGCT	Japan	103	41, 40%	IHC	Intensity score ≥ 1	NR	NR	7
Zhu X	2016	HCC	China	106	73, 69%	IHC	Final staining scores^c^ ≥ 5	OS (M)	≥ 5 years	8
Liu S	2019	CC	China	122	81, 66%	IHC	Final staining scores^a^ ≥ 3	OS (M), DFS (M)	≥ 5 years	7
Wei CY	2019	Melanoma	China	197	98, 50%	IHC	integrated optical density value(≥ median value)	OS (M), DFS (M)	≥ 5 years	7
Wang H	2018	Osteosarcoma	China	90	53, 58.9%	IHC	Final staining scores ^a^ > 5	NR	NR	8

*Abbreviations*: *GC* Gastric cancer, *BC* Breast cancer, *EC* Esophageal cancer, *EMC* Endometrial carcinoma, *EOC* Epithelial ovarian cancer, *ICC* Intrahepatic cholangiocarcinoma, *NSCLC* Non-small cell lung cancer, *TGTC* Testicular germ cell tumor, *HCC* Hepatocellular carcinoma, *CC* Cervical cancer; *OS* Overall survival, *DFS* Disease-free survival, *NR* Not report, *M* Multivariate analysis, *C* Curves, *IHC* Immunohistochemistry, *HR* Hazard ratio; ^a^ the intensity (intensity score: 0, negative; 1, weak; 2, moderate; 3, strong) and percentage of the total cell population (proportion score:0 < 10%; 10% ≤ 1 ≤ 33%; 34% ≤ 2 ≤ 66%; 67% ≤ 3 ≤ 100%); ^b^ staining intensity was divided into three steps (scores 0, 1, and 2) in the infiltrated region of each case. When score 0 was defined as negative and score 1 and score 2 were positive; ^c^TRIM44 staining was evaluated based on intensity scores (0, no staining; 1, weak staining; 2, moderate staining; and 3±, strong staining) and percentage scores (0, 0%; 1 ≤ 25%; 2, > 25%- ≤ 50%; 3, 50% > − ≤ 75%; 4, > 75%)

### The correlation between increased TRIM44 expression and OS

**Fig. 2:**
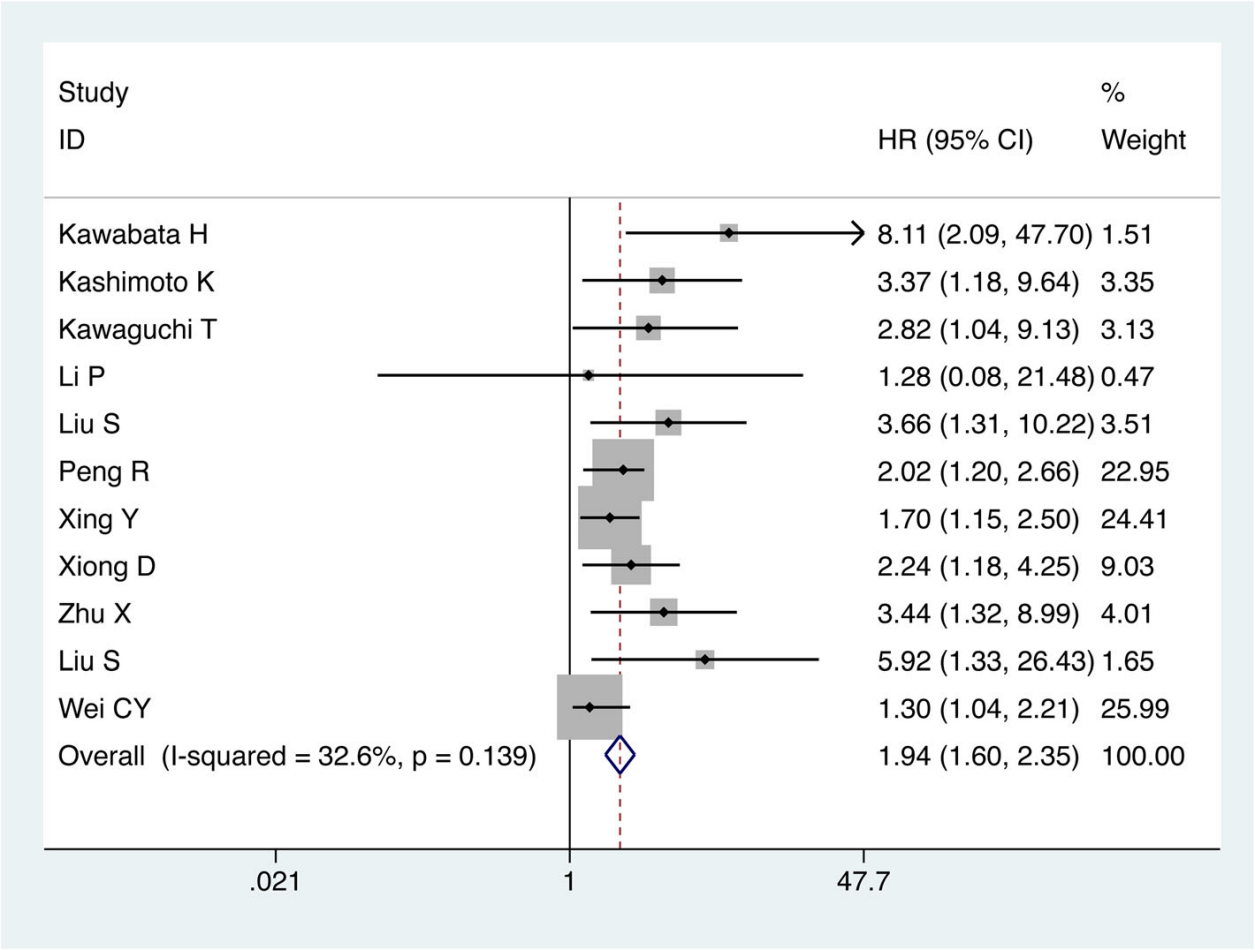
Forest plot of HR for the relationship between increased TRIM44 expression and OS

Eleven studies involving 1547 malignancy patients reported the HRs for OS. The pooled results for OS are displayed in Fig. 2. High expression of TRIM44 protein in malignant tissues was found to be strongly associated with poor OS (HR = 1.94, 95% CI: 1.60–2.35, *p < 0.0001*), and the heterogeneity test revealed a mild heterogeneity (I^2^ = 32.6%; P_Q_ = 0.139). The over-expression of TRIM44 could serve as a poor prognostic factor in human malignancies. In addition, for OS, the pooled HR values > 1 were consistently calculated in the subgroup meta-analysis stratified by the analysis type, sample size, and cutoff value; thus, indicating a significant difference among the two groups (Table 2).

**Table 2:** Pooled HR for OS according to the subgroup analysis

Categories	Studies(n)	Number of patients	HR (95% CI)	*p*-value	Heterogeneity		
					I^**2**^ (%)	P_Q_	Model
All	11	1547	1.94(1.60–2.35)	< 0.0001	32.6	0.139	Fixed
Cutoff value for high expression							
Final staining scores: 4 or 5	7	1118	2.03(1.42–2.19)	< 0.0001	40.9	0.119	Fixed
Final staining scores: 3	2	231	4.27(1.83–9.95)	0.001	0.0	0.604	Fixed
Others	2	198	2.10(1.44–3.06)	< 0.0001	0.0	0.571	Fixed
Analysis type							
Multivariate	10	1404	2.20(1.66–2.93)	< 0.0001	39.0	0.098	Fixed
Survival curves	1	143	8.66(1.10–68.22)	0.001	–	–	–
Sample size							
≥100	10	1479	2.29(1.68–3.12)	< 0.0001	44.9	0.060	Fixed
< 100	1	68	2.82(1.04–9.13)	0.041	–	–	–

### The correlation between increased TRIM44 expression and DFS

**Fig. 3:**
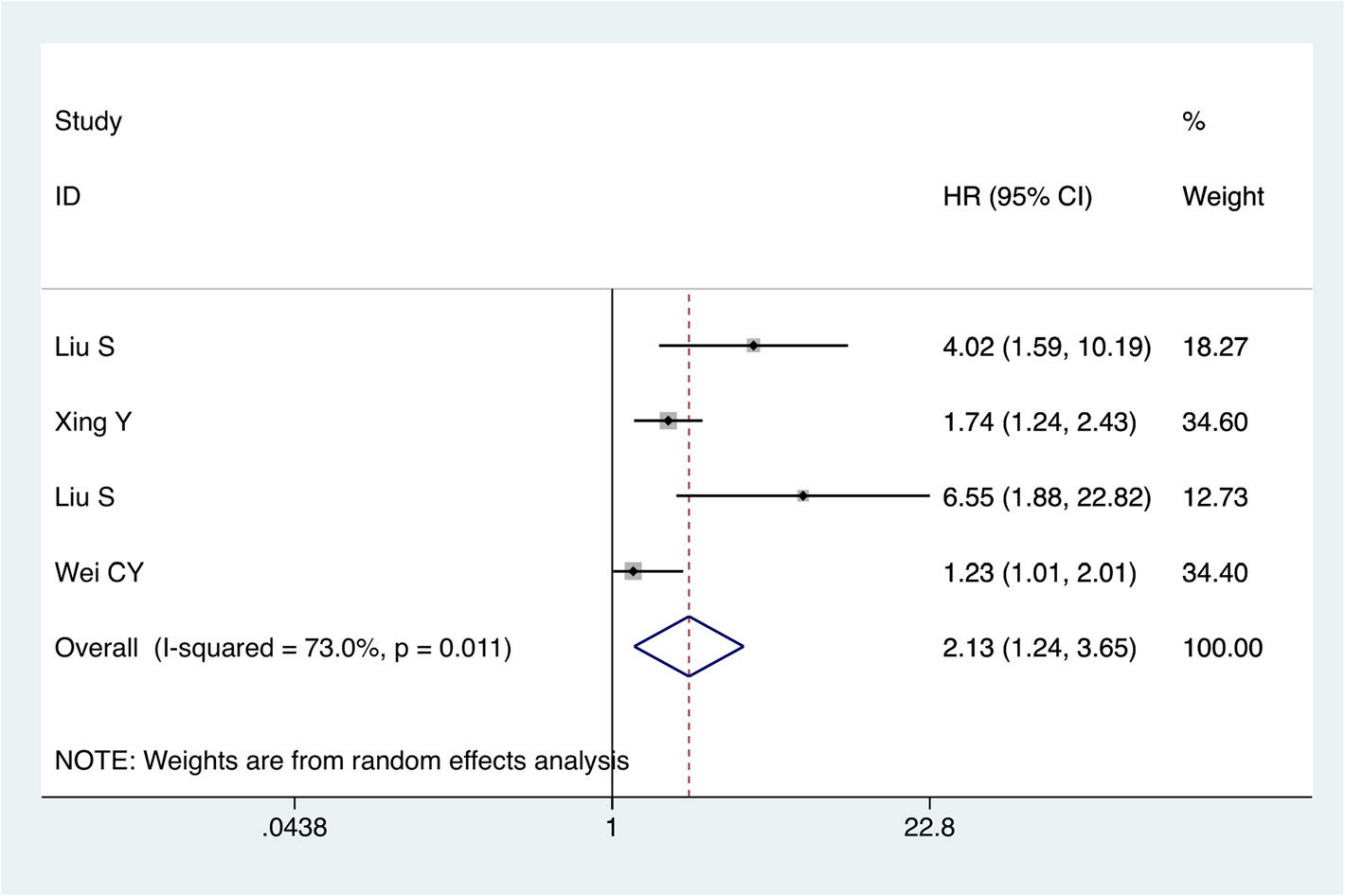
Forest plot of HR for the relationship between increased TRIM44 expression and DFS

Four studies involving 759 cases investigated the association between TRIM44 expression and DFS. Elevated expression of TRIM44 indicated an inferior DFS outcome, with a combined HR of 2.13 (95% CI 1.24–3.65, *p < 0.0001*). The combined result indicated that patients with elevated TRIM44 protein expression had a worse DFS rate compared to patients with lower TRIM44 protein expression (Fig. 3).

### The correlation between increased TRIM44 expression and clinicopathological parameters

The pooled odds ratios (ORs) were calculated to assess the risk of over-expression of TRIM44 protein under different clinicopathological features (Table 3). Tumors with unfavorable clinicopathological parameters were more frequently associated with overexpression of TRIM44 protein. These parameters included deeper tumor invasion (OR = 2.72, 95% CI: 1.73–4.30), poor tumor differentiation (OR = 1.78, 95% CI: 1.03–3.09), poor clinical stage (OR = 2.75, 95% CI: 2.04–3.71), distant metastasis (OR = 10.35, 95% CI: 1.01–106.24), lymph node metastasis (OR = 2.69, 95% CI: 1.71–4.24), and tumor recurrence (OR = 2.30, 95% CI: 1.34–3.95). However, there was no significant association between elevated TRIM44 expression and gender (OR = 1.00, 95% CI: 0.78–1.29, *p* = 0.990) or vascular invasion (OR = 2.43, 95% CI: 0.85–6.94, *p* = 0.097) in patients.

**Table 3:** Results of the meta-analysis of high TRIM44 and clinicopathological parameters

Clinicopathological parameter	Studies(n)	OR(95% CI)	*p*-value	Heterogeneity		
				I^**2**^ (%)	P_**Q**_	Model
Gender (male vs. female)	8	1.00(0.78–1.29)	0.990	0.0	0.699	Fixed
Recurrence (+ vs. -)	3	2.30(1.34–3.95)	0.002	14.2	0.312	Fixed
Tumor depth (T3–4 vs. T1–2)	4	2.72(1.73–4.30)	< 0.0001	0.0	0.488	Fixed
Lymph node metastasis (+ vs. -)	10	2.69(1.71–4.24)	< 0.0001	56.4	0.014	Random
Distant metastasis (+ vs. -)	3	10.35(1.01–106.24)	0.049	90.0	< 0.0001	Random
TNM stage (III-IV vs. I-II)	7	2.75(2.04–3.71)	< 0.0001	32.7	0.179	Fixed
Poorly/undifferentiated vs. well/moderately	6	1.78(1.03–3.09)	< 0.0001	62.8	0.020	Random
Vascular invasion (+ vs. -)	3	2.43 (0.85–6.94)	0.097	71.7	0.029	Random

### TRIM44 expression in different cancer types

**Fig. 4:**
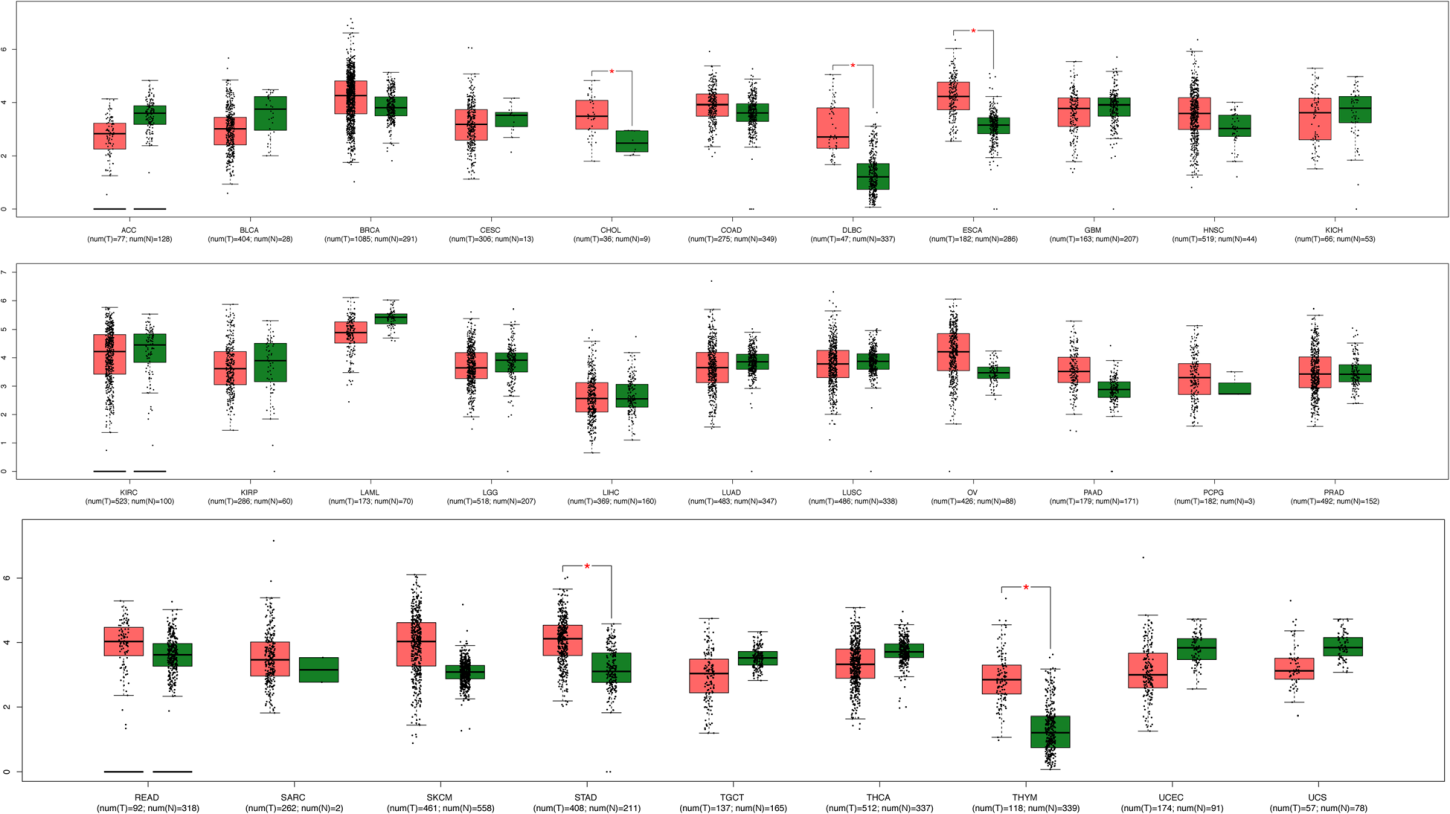
TRIM44 mRNA expression in different types of human malignancies

The expression level of TRIM44 mRNA from the GEPIA in different types of cancers was shown in Fig. 4. Among them, the expression of TRIM44 mRNA was significantly higher in malignant tissues than in the corresponding normal tissues, including CHOL, DLBC, ESCA, STAD, and THYM (Fig. 4).

### Validation of the prognostic value of TRIM44 in human tumors

**Fig. 5:**
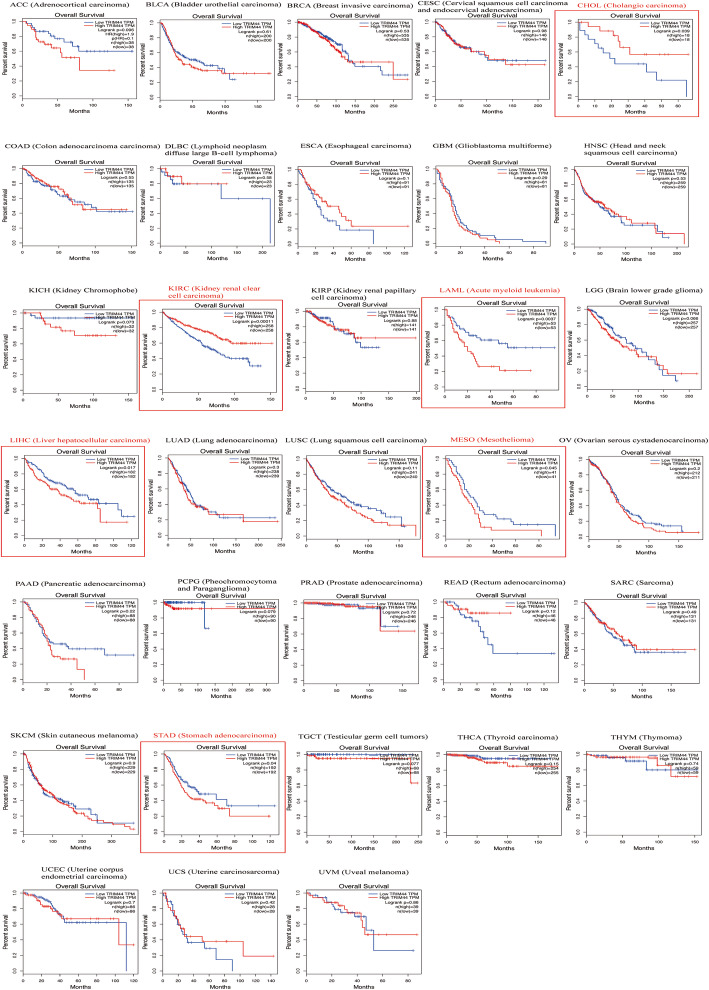
Kaplan-Meier plotter of OS for 33 types of malignant tumors

**Fig. 6:**
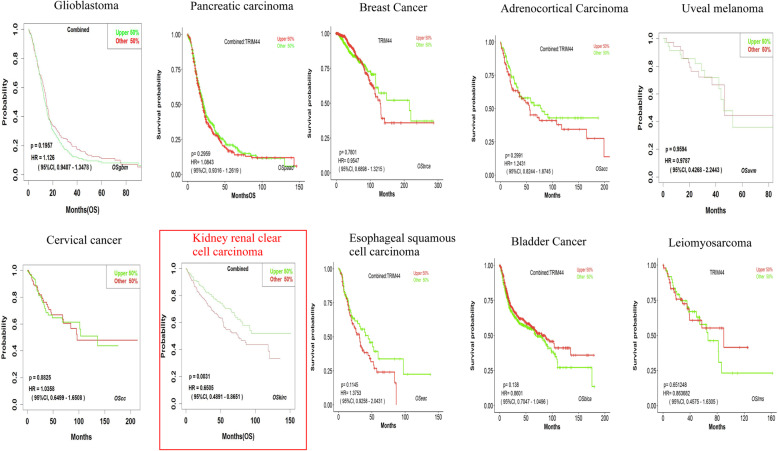
Kaplan-Meier plotter of OS in multiple analysis webservers

**Fig. 7:**
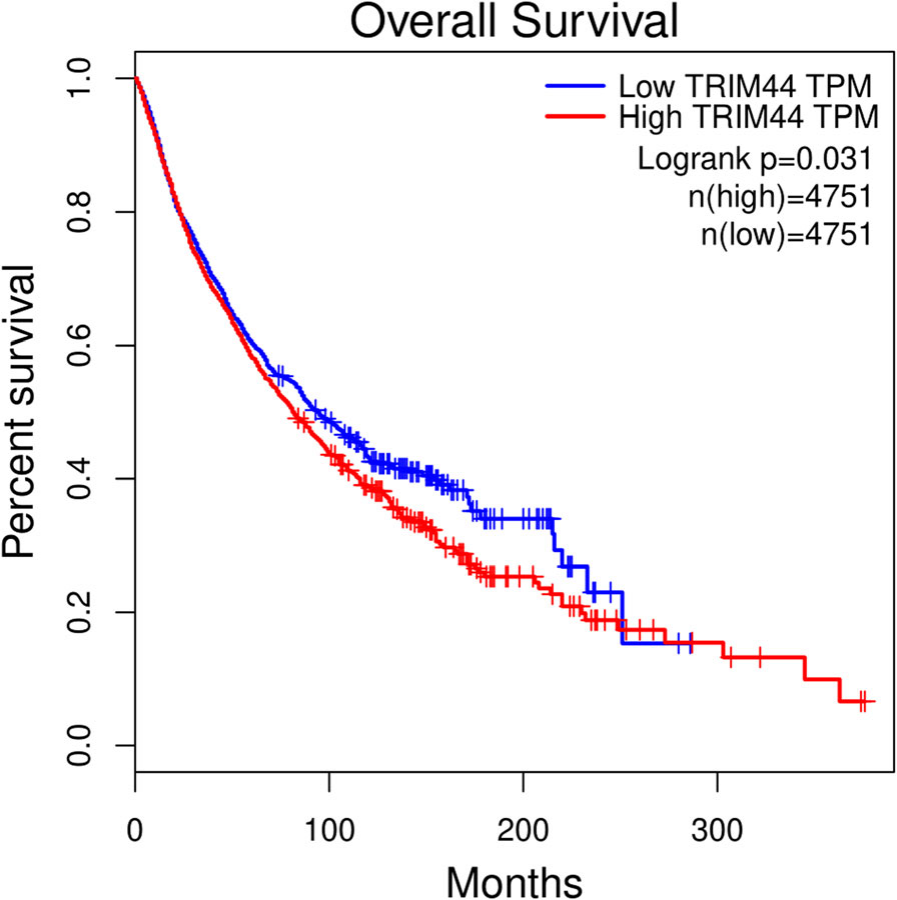
Kaplan-Meier plotter of OS for all cancer patients

In a survival analysis conducted through the GEPIA database, we found that high level of TRIM44 mRNA was shown to be significantly associated with unfavorable OS in patients with LAML, LIHC, MESO, and STAD (Fig. 5). However, for patients with CHOL or KIRC, a high level of TRIM44 mRNA in tumor samples indicated favorable OS in these cases (Fig. 5). And the significant prognostic value of TRIM44 mRNA in patients with KIRC was also displayed using OSkirc webserver (Fig. 6). When combined with all data from 33 different types of malignant tumors in GEPIA, the Kaplan-Meier analysis suggested that cancer patients with a high expression level of TRIM44 exhibited poorer OS, compared with cases expressing a low level of TRIM44 (Fig. 7).

**Fig. 8:**
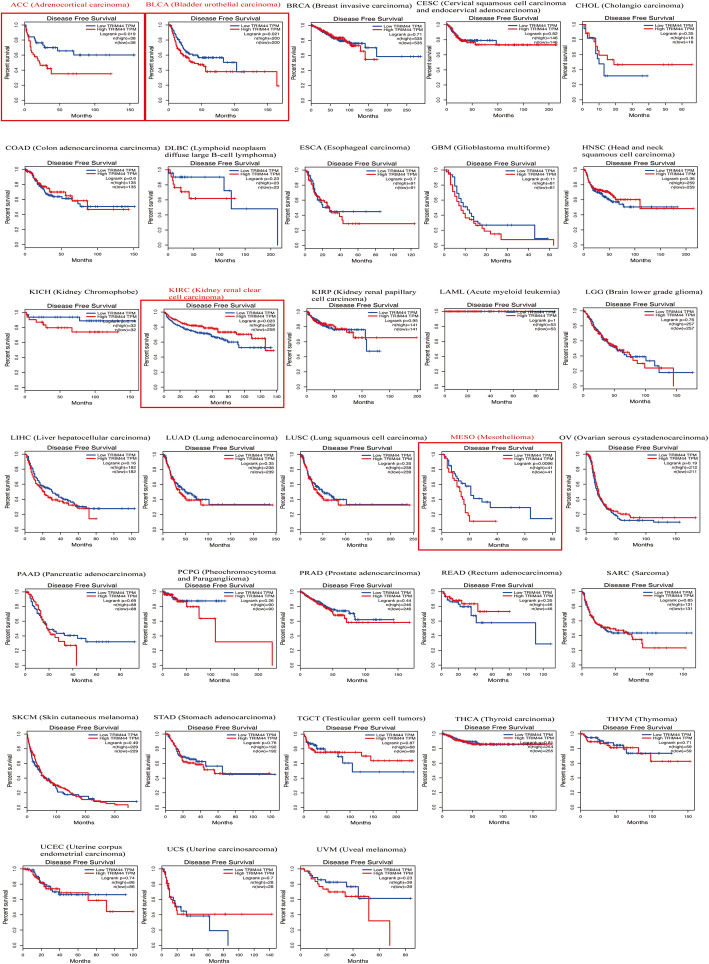
Kaplan-Meier plotter of DFS for 33 types of malignant tumors

**Fig. 9:**
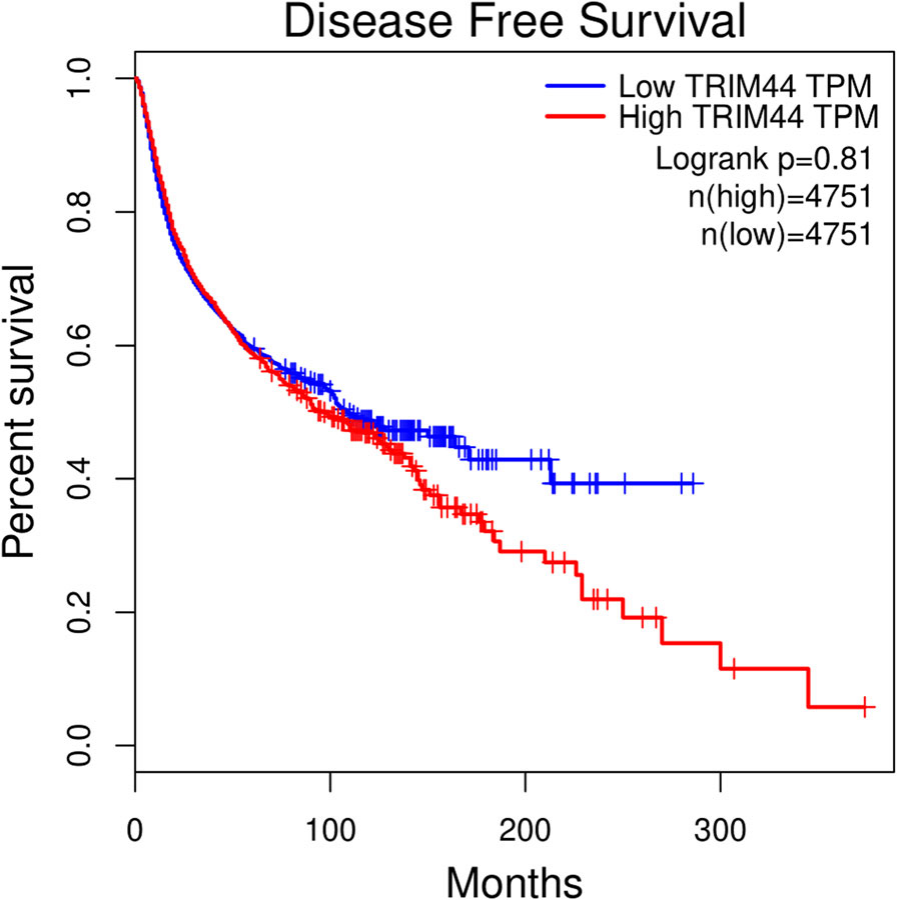
Kaplan-Meier plotter of DFS for all cancer patients

For the relationship between TRIM44 mRNA level and DFS in human tumors, overexpression of TRIM44 mRNA could be as an unfavorable prognostic biomarker for DFS in patients with ACC, BLCA, and MESO (Fig. 8), but as a favorable predictive factor for DFS in cases with KIRC (Fig. 8). And when combined with all tumor data from the GEPIA database, the pooled results showed that cancer patients with a high level of TRIM44 tend to have a poor DFS, although there was no statistical difference (Fig. 9).

### Publication bias

**Fig. 10:**
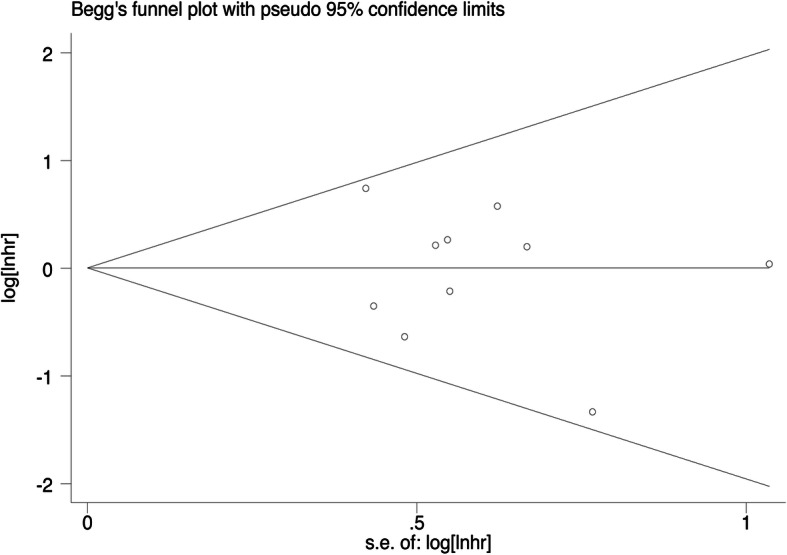
Publication bias assessment of TRIM44 expression and OS

The Begg’s visible plots are shown in Fig. 10, and the *p*-value of Begg’s test was 0.531 for OS. These results suggested that there was no publication bias in the present meta-analysis.

### Sensitivity analysis

**Fig. 11:**
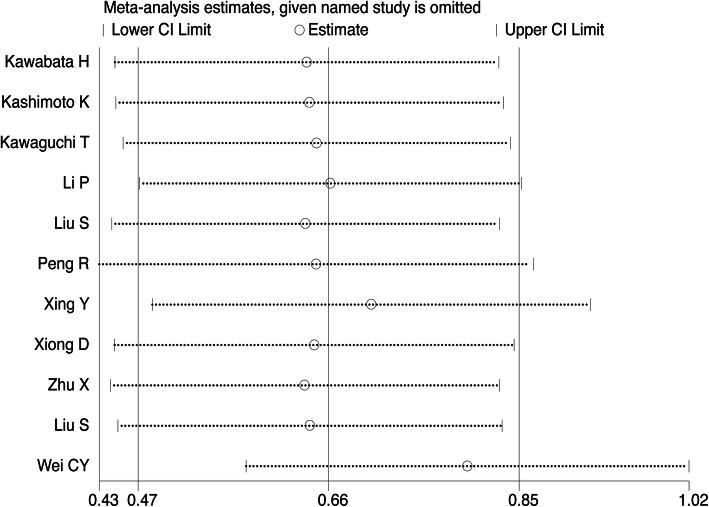
Sensitivity analysis of TRIM44 expression and OS

Figure 11 shows that the pooled results in this meta-analysis were relatively robust.

## Discussion

Ubiquitination is a post-translational protein modification that tags proteins for proteolytic degradation. It is involved in intercellular biological behavior, such as signal transduction, cell cycle regulation, DNA repair, antigen processing, and apoptosis [37–39]. RING finger ubiquitin E3 ligases have been previously reported to participate in cell-cycle regulation and carcinogenesis in malignancy [40–42]. Recent studies have suggested that some TRIM proteins, which contain a conserved RING finger, B-box, and coiled-coil domains, function as vital regulators of carcinogenesis [1, 43, 44]. These TRIM family proteins are associated with wide biological phenomena, including cell cycle regulation, transcriptional regulation, apoptosis, and carcinogenesis [45, 46].

TRIM-containing protein 44 (TRIM44: 11p13) contains B-box, coiled-coil domains, and a zinc-finger domain, which was discovered in ubiquitin hydrolases [6]. TRIM44 protein and mRNA have been reported to be significantly upregulated in cancer tissues compared to para-cancerous or normal samples. TRIM44 amplification is correlated with unfavorable prognosis and advanced clinicopathological parameters of malignancies [9, 12, 27]. Increased expression of TRIM44 could promote cell proliferation, migration, and invasion, whereas down-regulation of TRIM44 could significantly inhibit these pathologic features. TRIM44 may act as a cancer-promoting gene regulating deubiquitination and stabilization of oncogenes.

Tumor metastasis is a multistep process that starts with tumor migration and invasion through endothelial barriers via a process known as EMT, which is characterized by loss of cell-cell adhesion and increased cell motility [47–49]. Loss of E-cadherin expression, a hallmark of EMT, has been noted in many malignancies and is associated with increased metastatic potential [50, 51]. Cell motility, migration, and invasion are the malignant biological behaviors of cancer cells and the necessary factors for cancer metastasis. Knockdown of TRIM44 inhibited the invasion and migration of human NSCLC cells, and it was associated with the downregulation of mesenchymal markers (such as vimentin and N-cadherin) and upregulation of epithelial markers (such as E-cadherin) [10]. Overexpression of TRIM44 repressed E-cadherin expression and increased vimentin and N-cadherin expression in NSCLC cell lines [10]. Overexpression of TRIM44 induced EMT and increased the metastatic potential of lung cancer cells. In HCC cell lines, it was found that the ectopic expression of TRIM44 dramatically increased the expression of mesenchymal markers N-cadherin and vimentin, but it decreased the expression of epithelial marker E-cadherin; thus, suggesting that overexpression of TRIM44 could potentiate the EMT program [36]. Overexpression of TRIM44 has been shown to induce a similar change in hallmark characteristics of EMT in other cancers, such as ICC and HEC.

Uncontrolled cell proliferation is the biological characteristic of malignant tumors at the cellular level, and abnormal cell cycle regulation is closely related to uncontrolled cell proliferation [50]. TRIM44 expression positively affects the expression of cyclins and CDKs, suggesting that TRIM44 is involved in the regulation of cell cycle G1/s transformation [10, 36]. TRIM44 induced cell proliferation in vitro and tumor growth in vivo by accelerating the G1/S transition via the upregulation of cyclins and CDKs. Indeed, ectopic expression of TRIM44 promotes cell proliferation by accelerating the G1/S-phase transition in HCC. In colony formation assays, knockdown of TRIM44 in Huh7 cells significantly decreased the expression levels of cyclin D1 and cyclin E, which have been shown to play a crucial role in accelerating the G1/S-phase transition [36]. p21/p27 was discovered to function as a vital cyclin-dependent kinase inhibitor, and up-regulated expression of p21/p27 can inhibit cell entry into the S phase [52, 53]. Knock-down of TRIM44 in glioma cells induces an increase in p21/p27 expression,and then it inhibited cell division [54]. Further, the critical p21/p27 regulator AKT is inactivated after TRIM44 is knocked down, but it is activated in glioma cells that overexpress TRIM44 [54].

TRIM44 overexpression leads to high mTOR activity, which is consistent with observations of reduced mTOR signaling in cancer cell lines after siRNA knockdown of TRIM44 [12]. The phosphorylation of downstream mTOR substrates, including p-Akt (Ser473) and p-p70S6K (Thr389), in TRIM44-knockdown cells was markedly inhibited, indicating that TRIM44 functions upstream of the mTOR signaling pathway by phosphorylating mTOR. STAT3 participates in multiple biological behavior regulation, including cell proliferation, apoptosis, and tumorigenesis, and it has been reported to be involved in oncogene regulatory pathways, including AKT/mTOR [55–57].

TRIM44 could function as an E3 ubiquitin ligase and has ubiquitination function. TRIM44 could inhibit the role of AMPK in cells by degrading AMPK [6, 58]. AMPK has been reported to inhibit mTOR signaling. The upregulated TRIM44 reduced AMPK activity, thus relieving the inhibition of AMPK in the mTOR signaling pathway, and up-regulating the mTOR signaling from other mechanisms.

TRIM44-induced mTOR signaling, EMT, and cyclin/CDK upregulation was reversed by treatment with an inhibitor of the mammalian target of rapamycin (mTOR) [10]. Several previous studies have suggested that MAPK signaling pathways can induce EMT in cancer cells [11, 59, 60]. Inhibition of MAPK signaling by incubation with a signaling inhibitor significantly repressed ICC cell invasion and metastasis [32]. ERK1/2 has been reported to be involved in the regulation of EMT in ICC cells. TRIM44 could increase the activation of the AKT signaling pathway and activate ERK1/2; thus, suggesting that TRIM44 promotes EMT in ICC cells via the ERK-MAPK pathway.

Furthermore, overexpression of TRIM44 has been reported to be associated with inhibition of apoptosis in esophageal cancer [12]. Microarray analysis showed that TRIM44 knockdown is associated with the dysregulation of NUPR1, CDK19, CADM1, INHBA, TNFSF10, and DDIT4, which could normally activate the apoptotic cell pathways [31]. Bax and Bcl-2 are closely related to apoptosis. Elevated TRIM44 expression significantly repressed Bax and promoted Bcl-2 expression. Thus, TRIM44 has a vital role in inhibiting cellular apoptosis. NF-κB, functioning as a vital nuclear transcription factor, has been reported to be closely related to the inflammatory response, cellular apoptosis, and stress responses. NF-κB is the molecular target of some antitumor drugs [61]. The transcription factor NF-κB has been reported to inhibit apoptosis and to induce drug resistance in cancer cells [62]. Of note, it has been reported that TRIM44 promotes NSCLC development through activation of NF-κB signaling [13]. Previous studies have indicated that cIAP1, c-IAP2, and XIAP are the antiapoptotic transcriptional targets of NF-κB signaling [63]. A previous report has shown that the silencing of TRIM44 could decrease the c-IAP1, c-IAP2, and XIAP expression levels, especially in the presence of doxorubicin [36]. High expression of TRIM44 could enhance the resistance of HCC cells to doxorubicin via accelerating NF-κB activation. Increased NF-κB-mediated transcriptional activity was detected in TRIM44-transfected breast cancer cells [29].

Elevated TRIM44 protein expression enhanced proliferation and migration of TGCT cells, while TRIM44 protein knockdown repressed this biological behavior and promoted cell apoptosis.

Ki67 has been reported to be a promoter of cell proliferation [64]. TRIM44 modulates Ki67 expression and promotes HEC cell proliferation. Amplified TRIM44 expression was also discovered in melanoma tissues, and overexpression of TRIM44 is associated with a malignant phenotype of melanoma [35]. TRIM44 deubiquitinates and stabilizes TOLL-like receptor 4, which activates the AKT/mTOR pathway and induces cellular EMT. Moreover, miR-26b-5p is the upstream regulatory gene of TRIM44, which acts as a suppressor [35].

We believe that this study is the first meta-analysis to provide a systematic assessment of the prognostic value of TRIM44 in malignancies. Further, we have discussed the possible role of TRIM44 in tumor progression. Pooled results indicated that the TRIM44 protein level could act as a valuable prognostic marker in patients with malignancies. Malignancy patients with increased tissue TRIM44 expression had a significantly shorter OS and a lower DFS rate than patients with low TRIM44 expression. Furthermore, cancer patients with unfavorable clinicopathological parameters more frequently showed overexpression of TRIM44 protein. The GEPIA analysis results showed that TRIM44 was frequently overexpressed in multiple malignant tumors, and cancer patients with increased tissue TRIM44 expression had unfavorable prognosis. TRIM44 was involved in the malignant biological behavior of tumor cells and played an intersection role in the gene regulatory pathways.

However, there are several possible limitations of our research that may interfere with the generalizability of these conclusions. First, more samples need to be included to confirm the reliability of the conclusion. Second, all patients were from Asian countries, and studies including patients from other countries and races are required. Third, HRs and the corresponding 95% CIs were extracted from the survival curves and this might be less accurate than those directly obtained from the studies in multivariate analysis. Also, heterogeneity still exists in some results of clinicopathological characteristics.

## Conclusion

The present study demonstrated that the TRIM44 level is correlated with disease progression and prognosis in patients with malignancies. We found that TRIM44 is involved in the malignant biological behavior of tumor cells and it plays an intersection role in the gene regulatory pathways. Therefore, TRIM44 may be an important molecular marker for determining the malignant properties and an attractive therapeutic target for patients with malignancies.

## Data Availability

Meta-analysis is a secondary analysis, in which all data are fully available without restriction and all materials can be found in the included original studies.

## Abbreviations

TRIM44: Tripartite motif-containing 44
GEPIA: Gene Expression Profiling Interactive Analysis
TRIM: Tripartite motif
USPs: Ubiquitin-specific proteases
EMT: Epithelial-to-mesenchymal transition
NOS: Newcastle-Ottawa quality assessment scale
ACC: Adrenocortical carcinoma
BLCA: Bladder urothelial carcinoma
BRCA: Breast invasive carcinoma
CESC: Cervical squamous cell carcinoma and endocervical adenocarcinoma
CHOL: Cholangio carcinoma
COAD: Colon adenocarcinoma
DLBC: Lymphoid neoplasm diffuse large B-cell lymphoma
ESCA: Esophageal carcinoma
GBM: Glioblastoma multiforme
HNSC: Head and neck squamous cell carcinoma
KICH: Kidney chromophobe
KIRC: Kidney renal clear cell carcinoma
KIRP: Kidney renal papillary cell carcinoma
LAML: Acute myeloid leukemia
LGG: Brain lower grade glioma
LIHC: Liver hepatocellular carcinoma
LUAD: Lung adenocarcinoma
LUSC: Lung squamous cell carcinoma
MESO: Mesothelioma
OV: Ovarian serous cystadenocarcinoma
PAA: Pancreatic adenocarcinoma
PCPG: Pheochromocytoma and paraganglioma
PRAD: Prostate adenocarcinoma
READ: Rectum adenocarcinoma
SARC: Sarcoma
SKCM: Skin cutaneous melanoma
STAD: Stomach adenocarcinoma
TGCT: Testicular germ cell tumors
THCA: Thyroid carcinoma
THYM: Thymoma
UCEC: Uterine corpus endometrial carcinoma
UCS: Uterine carcinosarcoma
UVM: Uveal melanoma
GC: Gastric cancer
BC: Breast cancer
EC: Esophageal cancer
EMC: Endometrial carcinoma
EOC: Epithelial ovarian cancer
ICC: Intrahepatic cholangiocarcinoma
NSCLC: Non-small cell lung cancer
TGTC: Testicular germ cell tumor
HCC: Hepatocellular carcinoma
CC: Cervical cancer
OS: Overall survival
DFS: Disease-free survival
NR: Not report
M: Multivariate analysis
C: Curves
IHC: Immunohistochemistry
HR: Hazard ratio
OR: Odds ratio

## References

[1] Hatakeyama S (2011). TRIM proteins and cancer. Nat Rev Cancer.

[2] Leong PWF, Liew K, Lim W, Chow VTK. Differential display RT–PCR analysis of enterovirus-71-infected rhabdomyosarcoma cells reveals mRNA expression responses of multiple human genes with known and novel functions. 2004;295(1):147–59. 10.1006/viro.2002.1353 12033773

[3] Järvinen AK, Autio R, Kilpinen S, Saarela M, Leivo I, Grénman R, Mäkitie AA, Monni O. High-resolution copy number and gene expression microarray analyses of head and neck squamous cell carcinoma cell lines of tongue and larynx. Gene Chromosome Canc. 2008;47(6):500–9. 10.1002/gcc.20551 18314910

[4] Boutou E, Matsas R, Mamalaki A (2001). Isolation of a mouse brain cDNA expressed in developing neuroblasts and mature neurons. Brain Res Mol Brain Res.

[5] Yang B, Wang J, Wang Y, Zhou H, Wu X, Tian Z, Sun B. Novel Function of Trim44 Promotes an Antiviral Response by Stabilizing VISA. J Immunol. 2013;190(7):3613–9. 10.4049/jimmunol.1202507 23460740

[6] Urano T, Usui T, Takeda S, Ikeda K, Okada A, Ishida Y, Iwayanagi T, Otomo J, Ouchi Y, Inoue S. TRIM44 interacts with and stabilizes terf, a TRIM ubiquitin E3 ligase. Biochem Biophys Res Commun. 2009;383(2):268. 10.1016/j.bbrc.2009.04.010 19358823

[7] Weissman AM. Regulating protein degradation by ubiquitination. Immunol Today. 18(4):1997, 189–198. 10.1016/s0167-5699(97)84666-x 9136456

[8] Toby GG, Gherraby W, Coleman TR, Golemis EA (2003). A novel RING finger protein, human enhancer of invasion 10, alters mitotic progression through regulation of Cyclin B levels. Mol Cell Biol.

[9] Xiong D, Jin C, Ye X, Qiu B, Jianjun X, Zhu S, Xiang L, Wu H, Yongbing W (2018). TRIM44 promotes human esophageal cancer progression via the AKT/mTOR pathway. Cancer Sci.

[10] Xing Y, Meng Q, Chen X, Zhao Y, Liu W, Hu J, Xue F, Wang X, Cai L. TRIM44 promotes proliferation and metastasis in non-small cell lung cancer via mTOR signaling pathway. Oncotarget. 2016;7(21). 10.18632/oncotarget.8586 PMC5058694 27058415

[11] Zhang C, Liu LX, Dong ZR, Shi GM, Cai JB, Zhang PF, Ke AW, Yu JX, Zhou J, Fan J (2015). Up-regulation of 14-3-3zeta expression in intrahepatic cholangiocarcinoma and its clinical implications. Tumour Biol.

[12] Ong CA, Shannon NB, Ross-Innes CS, O'Donovan M, Rueda OM, Hu DE, Kettunen MI, Walker CE, Noorani A, Hardwick RH, Caldas C, Brindle K, Fitzgerald RC (2014). Amplification of TRIM44: pairing a prognostic target with potential therapeutic strategy. J Natl Cancer Inst.

[13] Luo Q, Lin H, Ye X, Huang J, Lu S and Xu L. Trim44 facilitates the migration and invasion of human lung cancer cells via the NF-κB signaling pathway. Int J of Clin Oncol. 2015;20(3):508–17. 10.1007/s10147-014-0752-9 25345539

[14] Wells G, Shea B, O'Connell J. The Newcastle-Ottawa scale (NOS) for assessing the quality of nonrandomised studies in meta-analyses. Ottawa Health Research Institute Web site. 2014;7(1).

[15] Tang Z, Li C, Kang B, Gao G, Li C, Zhang Z (2017). GEPIA: a web server for cancer and normal gene expression profiling and interactive analyses. Nucleic Acids Res.

[16] Dong H, Wang Q, Li N, Lv J, Ge L, Yang M, Zhang G, An Y, Wang F, Xie L, Li Y, Zhu W, Zhang H, Zhang M, Guo X (2019). OSgbm: An online consensus survival analysis web server for Glioblastoma. Front Genet.

[17] Zhang G, Wang Q, Yang M, Yao X, Qi X, An Y, Dong H, Zhang L, Zhu W, Li Y, Guo X (2020). OSpaad: An online tool to perform survival analysis by integrating gene expression profiling and long-term follow-up data of 1319 pancreatic carcinoma patients. Mol Carcinog.

[18] Yan Z, Wang Q, Sun X, Ban B, Lu Z, Dang Y, Xie L, Zhang L, Li Y, Zhu W, Guo X (2019). OSbrca: a web server for breast Cancer prognostic biomarker investigation with massive data from tens of cohorts. Front Oncol.

[19] Xie L, Wang Q, Nan F, Ge L, Dang Y, Sun X, Li N, Dong H, Han Y, Zhang G, Zhu W, Guo X (2019). OSacc: gene expression-based survival analysis web tool for adrenocortical carcinoma. Cancer Manag Res.

[20] Wang F, Wang Q, Li N, Ge L, Yang M, An Y, Zhang G, Dong H, Ji S, Zhu W, Guo X (2020). OSuvm: An interactive online consensus survival tool for uveal melanoma prognosis analysis. Mol Carcinog.

[21] Wang Q, Zhang L, Yan Z, Xie L, An Y, Li H, Han Y, Zhang G, Dong H, Zheng H, Zhu W, Li Y, Wang Y, Guo X (2019). OScc: an online survival analysis web server to evaluate the prognostic value of biomarkers in cervical cancer. Future Oncol.

[22] Xie L, Wang Q, Dang Y, Ge L, Sun X, Li N, Han Y, Yan Z, Zhang L, Li Y, Zhang H, Guo X (2019). OSkirc: a web tool for identifying prognostic biomarkers in kidney renal clear cell carcinoma. Future Oncol.

[23] Wang Q, Wang F, Lv J, Xin J, Xie L, Zhu W, Tang Y, Li Y, Zhao X, Wang Y, Li X, Guo X (2019). Interactive online consensus survival tool for esophageal squamous cell carcinoma prognosis analysis. Oncol Lett.

[24] Zhang G, Wang Q, Yang M, Yuan Q, Dang Y, Sun X, An Y, Dong H, Xie L, Zhu W, Wang Y, Guo X (2019). OSblca: a web server for investigating prognostic biomarkers of bladder Cancer patients. Front Oncol.

[25] Wang Q, Xie L, Dang Y, Sun X, Xie T, Guo J, Han Y, Yan Z, Zhu W, Wang Y, Li W, Guo X (2019). OSlms: a web server to evaluate the prognostic value of genes in Leiomyosarcoma. Front Oncol.

[26] Li P, Yin H, Meng F, Liu S, Ma R (2018). High TRIM44 expression in endometrial carcinoma is associated with a poorer patient outcome. Pathol Res Pract.

[27] Kashimoto K, Komatsu S, Ichikawa D, Arita T, Otsuji E (2012). Overexpression of TRIM44 contributes to malignant outcome in gastric carcinoma. Cancer Sci.

[28] Kawaguchi T, Komatsu S, Ichikawa D, Hirajima S, Nishimura Y, Konishi H, Shiozaki A, Fujiwara H, Okamoto K, Tsuda H. Overexpression of TRIM44 is related to invasive potential and malignant outcomes in esophageal squamous cell carcinoma. Tumour Biology the Journal of the International Society for Oncodevelopmental Biology & Medicine.2017;39(6):568835656. 10.1177/1010428317700409 28618928

[29] Hidetaka K, Kotaro A, Kazuhiro I, Ikuko S, Keiichi K, Takeshi F, Akihiko O, Toshiaki S, Kuniko H, Satoshi I. TRIM44 Is a Poor Prognostic Factor for Breast Cancer Patients as a Modulator of NF-κB Signaling. Int J Mol Sci. 2017;18(9):1931.

[30] Wang H, Zi-Ling F, Gong-Hao Z, Xin M. <em>TRIM44</em>, a crucial target of miR-410, functions as a potential oncogene in osteosarcoma. Oncotargets Therapy. 11:3637–47. 10.2147/OTT.S163163 PMC6016597 29950867

[31] Yamada Y, Takayama K, Fujimura T, Ashikari D, Obinata D, Takahashi S, Ikeda K, Kakutani S, Urano T, Fukuhara H. A novel prognostic factor TRIM44 promotes cell proliferation and migration, and inhibits apoptosis in testicular germ cell tumor. Cancer Sci. 2017;108(1):32–41. 10.1111/cas.13105 PMC5276827 27754579

[32] Peng R, Zhang PF, Zhang C, Huang XY, Ding YB, Deng B, Bai DS, Xu YP (2018). Elevated TRIM44 promotes intrahepatic cholangiocarcinoma progression by inducing cell EMT via MAPK signaling. Cancer Med.

[33] Liu S, Meng F, Ding J, Ji H, Lin M, Zhu J, Ma R. High TRIM44 expression as a valuable biomarker for diagnosis and prognosis in cervical cancer. Biosci Rep. 2019;39(3):BSR20181639. 10.1042/BSR20181639 PMC6400662 30792262

[34] Shuang L, Hexuan Y, Hongying J, Jiaqi Z, Rong M. Overexpression of TRIM44 is an independent marker for predicting poor prognosis in epithelial ovarian cancer. Exp Ther Med. 2018.16(06):3034–40. 10.3892/etm.2018.6541 PMC6125836 30214522

[35] Wei C, Wang L, Zhu M, Deng X, Wang D, Zhang S, Ying J, Yuan X, Wang Q, Xuan T. TRIM44 activates the AKT/mTOR signal pathway to induce melanoma progression by stabilizing TLR4. J Exp Clin Cancer Res. 2019;38(1):137. 10.1186/s13046-019-1138-7 PMC6437891 30922374

[36] Zhu X, Wu Y, Miao X, Li C, Yin H, Yang S, Lu X, Liu Y, Chen Y, Shen R, Chen X, He S (2016). High expression of TRIM44 is associated with enhanced cell proliferation, migration, invasion, and resistance to doxorubicin in hepatocellular carcinoma. Tumour Biol.

[37] Raboy B, Parag HA, Kulka RG. Conjugation of [125I] ubiquitin to cellular proteins in permeabilized mammalian cells: comparison of mitotic and interphase cells. EMBO J. 1986;5(5):863–69. 10.1002/j.1460-2075.1986.tb04296.x PMC1166875 3013620

[38] Fiore PPD, Polo S, Hofmann K. Opinion: When ubiquitin meets ubiquitin receptors: a signalling connection. 2003;4(6):491–7. 10.1038/nrm1124 12778128

[39] Wilkinson KD, Urban MK, Haas AL (1980). Ubiquitin is the ATP-dependent proteolysis factor I of rabbit reticulocytes. J Biol Chem.

[40] Burger MA. A Novel RING-Type Ubiquitin Ligase Breast Cancer-Associated Gene 2 Correlates with Outcome in Invasive Breast Cancer. Cancer Res. 2005;65(22):10401–12. 10.1158/0008-5472.CAN-05-2103 16288031

[41] Shabbeer S, Omer D, Berneman D, Weitzman O, Alpaugh A, Pietraszkiewicz A, Metsuyanim S, Shainskaya A, Papa MZ, Yarden RI. BRCA1 targets G2/M cell cycle proteins for ubiquitination and proteasomal degradation. Oncogene. 2013;32(42):5005–16. 10.1038/onc.2012.522 PMC3796024 23246971

[42] Ryu YS, Lee Y, Lee KW, Hwang CY, Maeng JS, Kim JH, Seo YS, You KH, Song B, Kwon KS. TRIM32 Protein Sensitizes Cells to Tumor Necrosis Factor (TNFα)-induced Apoptosis via Its RING Domain-dependent E3 Ligase Activity against X-linked Inhibitor of Apoptosis (XIAP). J Biol Chem. 2011;286(29):25729–38. 10.1074/jbc.M111.241893 PMC3138287 21628460

[43] Nisole S, Stoye JP, Sa BA. TRIM family proteins: retroviral restriction and antiviral defence. Nature Reviews Microbiology. 2005;3(10):799–808. 10.1038/nrmicro1248 16175175

[44] Di K, Linskey ME, Bota DA. TRIM11 is overexpressed in high-grade gliomas and promotes proliferation, invasion, migration and glial tumor growth. Oncogene. 2013; 32(42):5038–47. 10.1038/onc.2012.531 PMC3766389 23178488

[45] Ozato K, Shin D, Chang T, Morse HC. TRIM family proteins and their emerging roles in innate immunity. Nat Rev Immunol. 2008;8(11):849–60. 10.1038/nri2413 PMC3433745 18836477

[46] McNab FW, Rajsbaum R, Stoye JP, Garra AO. Tripartite-motif proteins and innate immune regulation. 2011;23(1):46–56. 10.1016/j.coi.2010.10.021 21131187

[47] Jou J, Diehl AM. Epithelial-mesenchymal transitions and hepatocarcinogenesis. J Clin Invest. 2010;120(4):1031–34. 10.1172/JCI42615 PMC2846072 20335655

[48] Acloque H, Adams MS, Fishwick K, Nieto MA, Bronner-Fraser M. Epithelial-mesenchymal transitions: the importance of changing cell state in development and disease. CELL. 2009;119(6):871. 10.1172/JCI38019 PMC2689100 19487820

[49] Connor KM, Hempel N, Nelson KK, Dabiri G, Gamarra A, Belarmino J, Van De Water L, Mian BM, Melendez JA. Manganese superoxide dismutase enhances the invasive and migratory activity of tumor cells. Cancer Res. 2007;67(21):10260–67. 10.1158/0008-5472.CAN-07-1204 17974967

[50] Zeisberg M, Neilson EG. Biomarkers for epithelial-mesenchymal transitions. J Clin Invest. 2009; 119(6):1429–37. 10.1172/JCI36183 PMC2689132 19487819

[51] Williams GH, Kai S (2012). The cell cycle and cancer. P Natl Acad Sci USA.

[52] Somasundaram K, Zhang H, Zeng YX, Houvras Y, Peng Y, Zhang H, Wu GS, Licht JD, Weber BL, Eldeiry WS (1997). Arrest of the cell cycle by the tumour-suppressor BRCA1 requires the CDK-inhibitor p21. Nature.

[53] Fillies T, Woltering M, Brandt B, Van Diest J, Werkmeister R, Joos U, Buerger H. Cell cycle regulating proteins p21 and p27 in prognosis of oral squamous cell carcinomas. Oncol Rep. 2007;17(2):355–9. 17203174

[54] Zhou X, Yang Y, Ma P, Wang N, Yang D, Tu Q, Sun B, Xiang T, Zhao X and Hou Z. TRIM44 is indispensable for glioma cell proliferation and cell cycle progression through AKT/p21/p27 signaling pathway. J of Neuro-Oncol. 2019;145(1–2). 10.1007/s11060-019-03301-0 31605296

[55] Yu H, Kortylewski M, Pardoll D. Crosstalk between cancer and immune cells: role of STAT3 in the tumour microenvironment. Nat Rev Immunol. 2007;7(1):41–51. 10.1038/nri1995 17186030

[56] Yu C, Meyer D, Campbell G, Larner A, Carter-Su C, Schwartz J and Jove R. Enhanced DNA-binding activity of a Stat3-related protein in cells transformed by the Src oncoprotein. Science. 269(5220):81–3. 10.1126/science.7541555 7541555

[57] Kortylewski M and Yu H. Stat3 as a Potential Target for Cancer Immunotherapy. J Immunother. 1995; 30(2):131–9. 10.1097/01.cji.0000211327.76266.65 17471161

[58] Pineda CT and Potts PR. Oncogenic MAGEA-TRIM28 ubiquitin ligase downregulates autophagy by ubiquitinating and degrading AMPK in cancer. Autophagy. 2015;11(5):844–6. 10.1080/15548627.2015.1034420 PMC4509443 25945414

[59] Santamaria PG, Nebreda AR (2010). Deconstructing ERK signaling in tumorigenesis. Mol Cell.

[60] Huang X, Ke A, Shi G, Zhang X, Zhang C, Shi Y, Wang X, Ding Z, Xiao Y, Yan J. αB-crystallin complexes with 14–3-3ζ to induce epithelial-mesenchymal transition and resistance to sorafenib in hepatocellular carcinoma. Hepatology. 57(6):2235–47. 10.1002/hep.26255 23316005

[61] Lai Y, Lin VTG, Zheng Y, Benveniste EN, Lin F (2010). The adaptor protein TRIP6 antagonizes Fas-induced apoptosis but promotes its effect on cell migration. Mol Cell Biol.

[62] Bentires-Alj M, Barbu V, Fillet M, Chariot A, Bours V (2003). NF-kappa B transcription factor induces drug resistance through MDR1 expression in cancer cells. ONCOGENE..

[63] Lin MT, Chang CC, Chen ST, Chang HL, Su JL, Chau YP and Kuo ML. Cyr61 expression confers resistance to apoptosis in breast cancer MCF-7 cells by a mechanism of NF-kappa B-dependent XIAP up-regulation. J Biol Chem. 2004;279(23):24015–23. 10.1074/jbc.M402305200 15044484

[64] Scholzen T, Gerdes J (2000). The Ki-67 protein: from the known and the unknown. J Cell Physiol.

